# mGWAS-Explorer 2.0: Causal Analysis and Interpretation of Metabolite–Phenotype Associations

**DOI:** 10.3390/metabo13070826

**Published:** 2023-07-05

**Authors:** Le Chang, Guangyan Zhou, Jianguo Xia

**Affiliations:** 1 Department of Human Genetics, McGill University, Montreal, QC H3A 0C7, Canada; le.chang@mail.mcgill.ca; 2 Institute of Parasitology, McGill University, Montreal, QC H9X 3V9, Canada; guangyan.zhou@mail.mcgill.ca

**Keywords:** mGWAS, metabolomics, causal inference, two-sample Mendelian randomization, semantic triples

## Abstract

Metabolomics-based genome-wide association studies (mGWAS) are key to understanding the genetic regulations of metabolites in complex phenotypes. We previously developed mGWAS-Explorer 1.0 to link single-nucleotide polymorphisms (SNPs), metabolites, genes and phenotypes for hypothesis generation. It has become clear that identifying potential causal relationships between metabolites and phenotypes, as well as providing deep functional insights, are crucial for further downstream applications. Here, we introduce mGWAS-Explorer 2.0 to support the causal analysis between >4000 metabolites and various phenotypes. The results can be interpreted within the context of semantic triples and molecular quantitative trait loci (QTL) data. The underlying R package is released for reproducible analysis. Using two case studies, we demonstrate that mGWAS-Explorer 2.0 is able to detect potential causal relationships between arachidonic acid and Crohn’s disease, as well as between glycine and coronary heart disease.

## 1. Introduction

The circulating metabolites can act as inputs, mediators or products in metabolic networks and play important roles in human health [1]. Over the past 15 years, growing applications of metabolomics in genome-wide association studies (mGWAS) have revealed a wealth of statistical associations between metabolites and genotypes [2,3,4,5], making mGWAS an important asset in the omics toolkits. Integrating these datasets has the potential to identify robust genetic underpinnings of diseases and traits with improved statistical power [2,3,5,6,7,8,9]. For instance, a recent large-scale meta-analysis integrating genetic associations for 174 metabolites from various platforms has significantly expanded our understanding of the genetic loci that impact these metabolite levels [2].

We developed mGWAS-Explorer to help researchers navigate the findings from different mGWAS studies [10]. Users can enter a list of genes, SNPs or metabolites and visually explore their known connections, as well as perform cross-phenotype association analysis. Over the past few years, causal inference techniques, such as Mendelian randomization (MR), have become integral to GWAS research, enabling causal estimations of exposures on outcomes, identifying novel risk factors, validating potential biomarkers and drug targets, as well as investigating gene–environment interactions [11,12,13,14,15]. MR uses genetic variants, known as instrumental variables, to infer potential causal relationships between exposures (e.g., metabolites) and outcomes (e.g., phenotypes). The utility of MR arises from the nature of genetic variants, which are randomly allocated at conception and remain fixed throughout an individual’s life, closely mirroring the random allocation of interventions in a randomized controlled trial. This unique characteristic strengthens the ability of MR to counteract confounding and reverse causation, which are two prevalent limitations in observational epidemiology [16]. The reliability of MR, however, depends on several assumptions, one of the most crucial being that the genetic variants should not influence the disease outcome via any pathway other than the exposure under investigation [17]. Many computational methods, databases and tools have been developed to support MR analysis [18,19,20,21]. Among them, two-sample Mendelian randomization (2SMR) emerges as a particularly useful method by enabling causal inference based on associations (genetic variant–exposure association and genetic variant–outcome association) from separate GWAS studies [22]. Bioinformatic resources such as IEU OpenGWAS and MR-Base [18,23] have been developed to streamline the process of causal inferences from an extensive collection of GWAS studies using 2SMR. They are becoming indispensable infrastructures for researchers in the field of genetic epidemiology.

MR has proven effective in estimating the causal effects of metabolites on diseases or other phenotypes by using metabolite quantitative trait loci (mQTLs) as genetic instrumental variables (IVs) [16,18,24]. For example, MR studies have identified the causal role of low-density lipoprotein cholesterol (LDL-C) in coronary artery disease (CAD), leading to the discovery of LDL-C-lowering drugs [25,26]. More recently, the phenome-wide Mendelian randomization (PheMR) has become a promising approach for investigating the potential causal associations between molecular phenotypes and a broad range of human traits and diseases [14,27,28]. PheMR analysis is a time-consuming, resource-intensive process and requires the careful selection of IVs [14]. Despite these advancements, a comprehensive and user-friendly bioinformatics tool for performing metabolome-wide MR mapping and interpretation has been lacking. Researchers usually need to use multiple tools coupled with script commands to obtain causal insights between genetically influenced metabolites and disease phenotypes. There is an unmet need for accessible bioinformatics tools to support MR analysis in mGWAS.

Interpreting causal assessments returned by MR methods remains a difficult task. One promising approach is to combine causal estimates with information derived from the literature, such as semantic triples (subject–predicate–object) [29]. This triangulation approach leverages literature-mined knowledge from resources such as Semantic MEDLINE Database (SemMedDB) and MELODI Presto to facilitate interpretations [30,31,32]. In addition to literature mining, the increased use of QTL analysis has notably broadened our capacity to explore complex genetic structures. Various molecular quantitative trait loci such as eGenes (eQTLs) or proteins (pQTLs) can provide important mechanistic links from genetic variants to phenotypes [33,34,35,36]. Integrating QTL data from various studies can increase statistical power and accuracy, as highlighted by two recent studies [37,38].

Here, we introduce mGWAS-Explorer 2.0 to address the evolving bioinformatics needs and challenges in mGWAS research. Compared to version 1.0, mGWAS-Explorer 2.0 contains several new features:

- Implemented a two-sample MR strategy to allow the investigation of causal relationships between >4000 metabolites and various phenotypes;
- Integration of semantic triples with eQTL and pQTL data to support functional annotation and mechanistic insights from MR results;
- Added a new “Joint Search” module that allows users to flexibly enter and search different molecules of interest;
- Enhanced data harmonization workflow and released the underlying mGWASR package to support reproducible analysis.

## 2. Materials and Methods

### 2.1. Knowledgebase Curation

The data source for the mGWAS summary statistics can be found in the publication of version 1.0 of mGWAS-Explorer [10]. The eQTL data from 49 tissues and pQTL data from blood were obtained from the Genotype–Tissue Expression (GTEx) project and QTLbase [39,40]. The complete GWAS summary statistics of the disease outcome were based on the Application Programming Interface (API) service of the IEU OpenGWAS [23].

### 2.2. Methods for MR Analysis

The statistical methods for pre-processing and MR analysis are based on the TwoSampleMR and MRInstruments R packages [18]. The pre-processing procedure facilitates the acquisition of independent instrumental variables by performing linkage disequilibrium (LD) clumping. In cases where the SNP query is absent in the outcome GWAS, we identify a proxy SNP in LD with the input SNP, utilizing the 1000 Genomes Project phase 3 data as a reference [41]. A crucial aspect of the analysis is harmonizing exposure and outcome data to make sure that the effects of the SNP on exposure and outcome are associated with the same allele. Three options are available: (i) assume all alleles are on the forward strand; (ii) infer the forward strand alleles based on allele frequency; (iii) adjust the strand for non-palindromic SNPs while excluding all palindromic SNPs.

Our approach incorporates 18 distinct MR methods together with support for heterogeneity and horizontal pleiotropy testing. In particular, the heterogeneity test is based on Cochran’s Q test, while the horizontal pleiotropy test is conducted using Egger regression. These tests allow a comprehensive understanding of the potential biases within the MR analysis and promote robust and reliable results.

### 2.3. Pre-Computed Phenome-Wide MR

A comprehensive collection of 1825 SNPs associated with 1016 distinct metabolites was derived from five recent mGWAS datasets [2,4,42,43,44]. We first selected SNPs that were associated with any metabolites with a *p*-value threshold of 5 × 10^−8^ in at least one of the five studies. Because of intricate LD patterns of SNPs located within the human major histocompatibility complex (MHC) region (chr6: from 26 Mb to 34 Mb), we excluded both SNPs and the associated metabolites within that region. Finally, we performed LD clumping for the IVs to identify independent SNPs for each metabolite using a threshold of r^2^ < 0.001. A total of 1544 IVs related to 825 metabolites were retained. We used 2SMR to comprehensively evaluate the potential causal impact of these 825 metabolites on 236 distinct phenotypes, which comprised an array of diseases and associated risk factors.

### 2.4. Semantic Triples

The semantic triples are queried using the API service of the MELODI Presto [32]. MELODI Presto enables the exploration of enriched literature data corresponding to specific search terms and the identification of potential intermediate disease mechanisms among term lists. The SemMedDB [31] serves as a repository for semantic predications, including subject–predicate–object triples.

### 2.5. R Package

The underlying analysis is based on the mGWASR package available on GitHub (https://github.com/xia-lab/mGWASR (accessed on 27 May 2023)). The R package includes detailed vignettes for step-by-step analysis. To guarantee that the identical results will be generated, the R package and the web server have been thoroughly tested.

## 3. Results

### 3.1. Two-Sample Mendelian Randomization (2SMR)

The growing number of mGWAS makes it possible to systematically investigate the potential causal relationships between metabolites (targeted) or metabolite features (untargeted) and human diseases. To facilitate this process, we implemented a “MR Analysis” module to support 2SMR analysis between >4000 metabolites and various phenotypes.

On the data upload page of this module, users first specify the metabolites (exposure) and disease (outcome) of interest. The mGWAS-Explorer 2.0 will automatically identify SNPs significantly associated with the metabolites from our curated mGWAS data and subsequently extract these instrumental SNPs associated with the disease outcome available in the IEU OpenGWAS database [23]. After acquiring summary statistics for both exposure and outcome, users need to harmonize data to ensure consistency in genetic instruments, effect sizes and effect alleles. The parameter page allows users to perform LD clumping or pruning, retaining only independent genetic variants for MR estimation [18]. Our platform currently offers 18 distinct MR analysis methods for causal effects estimation, including MR–Egger, weighted median, inverse variance-weighted methods, etc. [18]. Additionally, mGWAS-Explorer 2.0 automatically performs sensitivity assessments and heterogeneity tests to evaluate potential violations of MR assumptions and the robustness of causal estimates. Upon completion, the MR results are displayed in a summary table together with four types of graphical outputs. 

The summary table provides a grouped display containing the outputs from MR analysis, heterogeneity tests and horizontal pleiotropy tests. The MR analysis results within the table are organized to show the selected SNPs used as instrumental variables, along with their corresponding causal effect estimates, standard errors and *p*-values. The summary table also displays the outcomes of heterogeneity tests, detailing the Cochran’s Q statistic along with its degrees of freedom and the *p*-value. The Q statistic aids in determining if the variation seen in effect sizes is a result of randomness or actual heterogeneity. Further, the table displays the results of the horizontal pleiotropy tests. Key values such as the MR–Egger regression intercept and its corresponding *p*-value are presented. These metrics are crucial for detecting horizontal pleiotropy, which occurs when the SNPs have additional effects on the outcome aside from their impact on the exposure. 

Four types of plots are generated to visually represent the data and facilitate intuitive interpretation. (i) The forest plot provides a visual summary of the individual causal effect estimates from each SNP, with their corresponding confidence intervals contributing towards the overall MR estimate. This effectively allows for the evaluation of both the direction and magnitude of the causal effect and heterogeneity among variants. (ii) The scatter plot displays the causal effect estimates from each SNP against their respective associations with the exposure. This gives a graphical representation of the MR assumptions to aid in detecting potential outlier SNPs. (iii) The funnel plot provides a unique perspective by plotting the precision of individual variant estimates against their corresponding causal effect estimates. Users can visually identify asymmetry that might suggest the presence of directional pleiotropy or outliers. (iv) Lastly, the plot for leave-one-out analysis graphically demonstrates the robustness of the MR findings. It illustrates how the overall MR estimate varies when individual SNPs are excluded from the analysis to help identify influential SNPs that may unduly skew the results. These diagnostic plots allow users to thoroughly evaluate the MR results. Users have the option to customize these plots in terms of format, resolution or size for downloading purposes.

### 3.2. Pre-Computed Phenome-Wide MR

To facilitate the exploration of the potential causal relationships between metabolites and a range of phenotypes, we conducted a phenome-wide MR analysis based on five recent mGWAS [2,4,39,43,44] and identified 1825 SNPs associated with 1016 metabolites. After removing metabolites and SNPs by using the selection criteria (see Section 2), 1544 SNPs and 825 metabolites were kept as instrumental variables for MR analysis. We performed 2SMR to systematically assess the causal effects of these 825 metabolites on 236 phenotypes, including diseases and disease-related risk factors.

**Figure 1:**
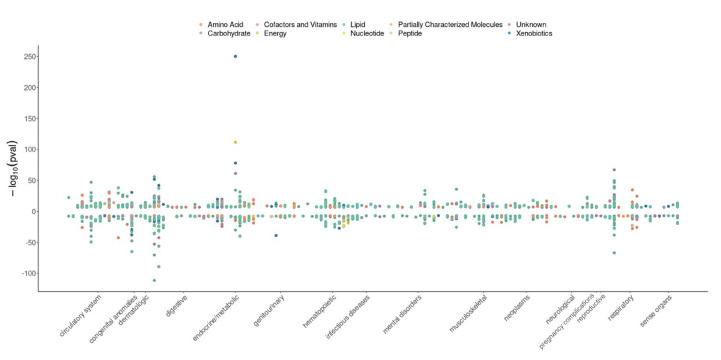
Miami plot showing significant phenome-wide Mendelian randomization results of metabolites. The *x* axis is the traits sorted according to a meaning set of biological categories (for example, circulatory system, digestive traits). The *y* axis represents the −log_10_ *p*-value of the MR results; MR results with positive effects (increased level of metabolites associated with increasing the phenotype risk) are represented on the top half of the plot, while MR results with negative effects (decreased level of metabolites associated with increasing the phenotype risk) are shown on the bottom half of the plot. The color indicates metabolite super pathways. The significant *p*-value cut-off for MR results is 2.57 × 10^−7^ at a Bonferroni-corrected threshold, 0.05/(825 × 236).

Our analysis identified 1243 metabolite–trait associations with significant MR evidence (*p* < 2.57 × 10^−7^ at a Bonferroni-corrected threshold, 0.05/(825 × 236)) (Figure 1 and Supplementary Table S1). We also conducted sensitivity analyses, including MR–Steiger filtering [45], to test for reverse causality and heterogeneity analyses [46] for metabolites that have multiple IVs. Our MR results revealed significant associations between metabolites and various disease categories, including many new associations that have not yet been reported before.

### 3.3. Triangulating Evidence from Semantic Triples

Integrating evidence from different studies could minimize the sources of bias and obtain more trustworthy answers. The basic concept is that if the results from different sources all lead to the same conclusion, the confidence in the findings increases. This strategy has been referred to as ‘triangulation’ and has gained increasing attention in epidemiology research for causal inference [30]. Driven by this concept, we have leveraged the MELODI Presto method to enable triangulation of the causal estimates from MR with millions of semantic triples curated from the literature [26]. 

In the result page of the MR module, users can retrieve the semantic triples (subject–predicate–object) associated with the exposure (metabolite) and the outcome (disease) to identify overlapping enriched terms, namely, the object from the exposure query overlaps with a subject from the outcome query [32]. The query usually takes a few seconds and returns the results in a data table. Alternatively, users can explore the results using a network diagram. 

### 3.4. Enabling Joint SNP/Metabolite Analysis

Recent studies have shown that leveraging SNPs and metabolite data has the potential to reveal associations beyond traditional metabolic pathways [47]. Therefore, we have added a new “Joint Search” module that enables users to input SNPs, metabolites or both simultaneously.

For metabolite input, users can enter various identifiers such as compound names, Human Metabolome Database (HMDB) IDs or Kyoto Encyclopedia of Genes and Genomes (KEGG) IDs. Users can then choose from multiple mapping options, such as linking metabolites to SNPs based on statistical associations, associating metabolites to genes through knowledge-based mappings or connecting metabolites to diseases.

The SNP input accepts rsID. Four different mapping options are available to link SNPs to metabolites, genes (either the nearest gene or eQTL), proteins (using pQTL) or diseases. Moreover, users can filter SNP to metabolite mappings based on specific biofluid or population data. The biofluids cover blood, urine, cerebrospinal fluid (CSF), saliva, as well as mitochondria, while the populations include European, American, Hispanic, Middle Eastern and South Asian groups.

The search results are networks representing complex associations between SNPs and metabolites mediated by other molecules. To further tailor the generated networks, users can apply filters based on network topology metrics such as node degree and betweenness, shortest path calculations, etc.

### 3.5. Improving Transparency/Reproducibility through Releasing mGWASR Package

Transparent data processing and analysis procedures are essential for reproducible research [48]. To support this direction, we have added a Result Download page in each module to allow users to obtain all results tables and images generated during the analysis, as well as the R command history. The underlying R functions of mGWAS-Explorer 2.0 are released as the mGWASR package (https://github.com/xia-lab/mGWASR (accessed on 27 May 2023)). We anticipate that the R package and the R command history will enable users to track each stage of their analysis in an easily sharable and reproducible format (i.e., R script). Additionally, we have migrated all our frequently asked questions (FAQs) to the OmicsForum (https://omicsforum.ca (accessed on 27 May 2023)) to better engage with our user communities.

### 3.6. Case Studies

#### 3.6.1. Crohn’s Disease Case Study

**Figure 2:**
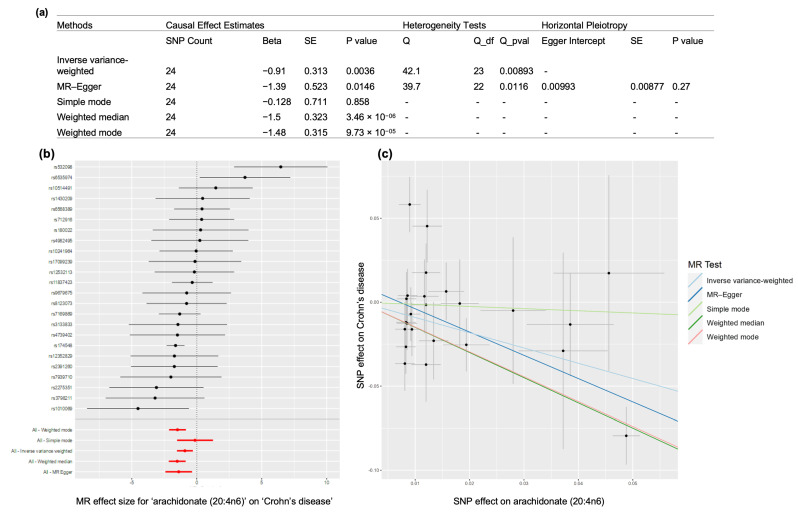
Assessment of causal effects of arachidonic acid levels on Crohn’s disease. (**a**) A summary table displaying the results of MR analysis, heterogeneity and horizontal pleiotropy tests; (**b**) a forest plot comparing the causal effects calculated using the methods including all the SNPs (illustrated by red color) to using each SNP separately (illustrated by black color). (**c**) a scatter plot showing the relationships between SNP effects on arachidonic acids against the SNP effects on Crohn’s disease, with slope indicating the causal association.

Crohn’s disease is a complex disease that causes chronic inflammation of the gastrointestinal tract. Previous studies have suggested that arachidonic acid has a causal effect on Crohn’s disease through colocalization analysis [49]. Therefore, we sought to investigate the potential causal effect of the arachidonic acid on Crohn’s disease using the summary statistics for both traits in mGWAS-Explorer 2.0 [4,50]. Using 24 independent genetic instruments (i.e., SNPs), the results of four commonly used MR methods (inverse variance-weighted, MR–Egger, weighted median estimator and weighted mode estimator) consistently illustrate that the decrease in arachidonic acid levels had a causal effect on Crohn’s disease (Figure 2 and Figure S1), which is consistent with the findings reported by Chu et al. [49]. This case study highlights how users can easily perform MR analysis by leveraging our comprehensive knowledgebase of mGWAS summary statistics as well as an easy-to-use interface to test the hypothesis of the plausible causal roles of metabolites on diseases. 

#### 3.6.2. Coronary Heart Disease Case Study

**Figure 3:**
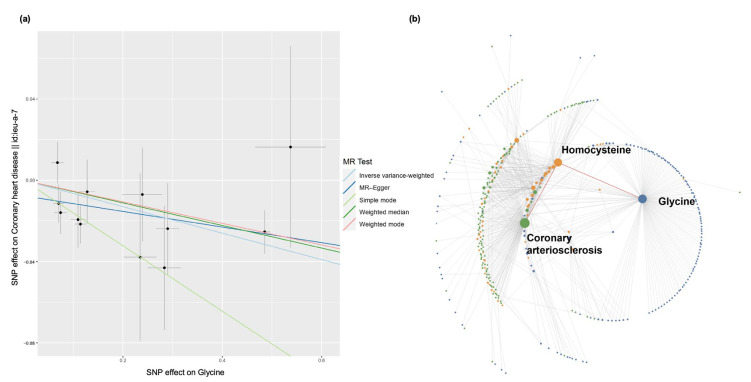
Triangulation of MR results and literature evidence on the effects of glycine on coronary heart disease case study. (**a**) A scatter plot showing the relationships between SNP effects on glycine against the SNP effects on coronary heart disease, with slope indicating the causal association; (**b**) a network of semantic triples (subject–predicate–object) from evidence from the literature between “glycine” and “coronary heart disease”. Each node represents either an exposure subject (blue), an outcome object (green) or an overlapping enriched element (orange), where the object of a triple from the exposure query overlaps with a subject of a triple from the outcome query. Each edge is a “predicate” connecting two semantic elements. The path between *glycine*, *homocysteine* and *coronary arteriosclerosis* is highlighted in orange.

To demonstrate triangulating casual inference from MR with evidence from the literature, we used glycine and coronary heart disease as an example to explore the semantic evidence connecting the metabolite and the disease. Figure 3a shows the causal associations between SNP effects on glycine against the SNP effects on the coronary heart disease. Genetic predisposition to higher glycine levels is associated with lower risk of coronary heart disease. Figure 3b displays the semantic-triples connections between glycine and coronary heart disease after searching for enriched overlapping terms. A total of 73 overlapping terms was identified, including homocysteine [51,52,53], ethanol [54,55] and TNF protein [56,57]. In the case of homocysteine, “homocysteine—PREDISPOSES—Coronary Arteriosclerosis” is the most enriched semantic triple on the outcome side (*p*-value: 4.38 × 10^−120^), whereas “Glycine—INTERACTS_WITH—homocysteine” has a *p*-value of 8.3 × 10^−6^. Therefore, we can hypothesize that the protective effect of glycine on coronary heart disease may be due to the interactions with homocysteine. 

### 3.7. Comparison with Other Tools

Table 1 compares mGWAS-Explorer 2.0 with its previous version and several other web-based tools, including EpiGraphDB [29], The Molecular Human [58] and MR-Base [18]. EpiGraphDB is a graph database and analytical platform containing comprehensive epidemiological and biomedical relationships, including pre-computed MR causal estimates, drugs, pathways, evidence from the literature, ontology information, etc. The Molecular Human focuses on providing a comprehensive characterization of the molecular interactions using the integrated multi-omics data from 18 different platforms. MR-Base is an integrated platform that automates the two-sample MR analysis with a web interface, API and R package, which incorporates a database of complete GWAS summary statistics. In comparison, mGWAS-Explorer 2.0 supports both linking multi-omics with diseases and performing MR analysis to identify metabolites with causal impacts on the diseases in the context of mGWAS. 

## 4. Discussion

Systematic causal inferences between modifiable risk factors and complex traits remain challenging in human genetics [14,59,60]. We have developed mGWAS-Explorer 2.0, which integrates published mGWAS summary statistics with analytical methods and visualization, with a particular focus on understanding the relationships between genetic variants, metabolites and diseases.

The main approach for causal estimates is based on 2SMR. However, 2SMR relies on strict assumptions [61]. To minimize the violations and help obtain valid causal estimates, we have implemented two types of protections—algorithmic and data protections. Algorithmic protections include two strategies: (i) Multiple IVs: leveraging multiple independent genetic variants as IVs can help mitigate the effects of individual genetic variants violating the MR assumptions. Techniques such as weighted median or MR–Egger regression can account for potential violations and yield more reliable causal estimates. (ii) Sensitivity analyses: conducting various sensitivity analyses can aid in identifying and evaluating the impact of potential MR assumption violations. For instance, MR–Egger regression can detect whether directional horizontal pleiotropy drives the results of an MR analysis. Data protections involve the following measures. (i) Large and well-characterized cohorts: utilizing data from sizable, well-characterized cohorts with high-quality genotyping and phenotyping information can minimize measurement errors and enhance the precision of causal estimates. This can help reduce violations of the relevance and independence assumptions. (ii) Data harmonization: by ensuring consistency in exposure and outcome definitions across the studies, potential biases stemming from varying definitions or data collection methods can be diminished.

One of the key characteristics of clinical phenotypes and disease relationships is their inherent complexity and diversity, with a single clinical phenotype potentially associated with multiple diseases and a single disease often linked with multiple phenotypes. The 2SMR approach used by mGWAS-Explorer 2.0 can tackle this scenario effectively, allowing for the investigation of whether a change in a metabolite concentration is causally related to each disease or phenotype individually. Importantly, our approach utilizes summary statistics from large-scale GWAS, providing robust, population-based evidence that is less prone to confounding bias.

The capabilities of mGWAS-Explorer 2.0 extend beyond traditional GWAS and molecular QTL analysis software. As highlighted in the studies by Shariatipour et al. [37,38], meta-analysis of QTL is usually conducted using a combination of several tools. In contrast, mGWAS-Explorer 2.0 provides a single platform to support causal analysis for more than 4000 metabolites and various phenotypes. It also enables result interpretation within the context of known molecular interactions as well as semantic triples based on literature mining. Such a streamlined process allows for the efficient, in-depth exploration of the data, facilitating novel hypothesis generation. 

Our first case study investigated the causal role of arachidonic acid (AA) on Crohn’s disease (CD) using MR. AA belongs to omega-6 polyunsaturated fatty acids, and free AA enhances and modulates type 2 immune response, which is crucial for resistance to allergens and parasites [62]. In our analysis, the negative causal effect of AA on CD is consistent with previous studies where CD patients had lower levels of AA [63,64]. However, more studies are needed to understand the mechanisms underlying the association. 

The second case study highlights the protective role of glycine on coronary heart disease (CHD), which agrees with the findings from the MR study by Wittemans et al. [65]. In the semantic triples analysis, “Glycine—INTERACTS_WITH—homocysteine” and “homocysteine—PREDISPOSES—Coronary Arteriosclerosis” present an example of how to obtain possible mechanisms from the literature after MR analysis. A high homocysteine level is strongly associated with the prevalence of CHD. The role of homocysteine on CHD is explained by its negative effects on vascular endothelium and smooth muscle cells [66]. On the other hand, it was reported that intracellular concentrations of homocysteine were lowered after 24 h of co-incubation with glycine [53], although the mechanism of how glycine lowers the homocysteine concentrations is not clear.

While mGWAS-Explorer 2.0 has been developed primarily for human studies, it can be extended to support a wider range of organisms. This is currently limited by the availability of comprehensive genomic and metabolic datasets for these organisms. Therefore, an important future effort will be collecting and integrating high-quality data from other species, such as plants [67].

## 5. Conclusions

We developed mGWAS-Explorer 2.0 to allow researchers to investigate potential causal relationships between metabolites and various phenotypes. By leveraging two-sample MR, together with text mining and molecular networks for functional interpretations, mGWAS-Explorer 2.0 has addressed a critical gap in the post-GWAS era [68]. The utilities are demonstrated in two case studies. We expect that mGWAS-Explorer 2.0 will play an important role in helping elucidate the etiology of disease with the growing number of published GWAS data.

## Figures and Tables

**Table 1:** Comparison of the main features of mGWAS-Explorer (version 1.0–2.0) with other web-based tools. Symbols used for feature evaluations: ‘√’ for present, ‘−’ for absent and ‘+’ for a more quantitative assessment (more ‘+’ symbols indicate better support).

Tool Name	mGWAS-Explorer		EpiGraphDB	The Molecular Human	MR-Base
	2.0	1.0			
**Data input and processing**					
Metabolite	√	√	√	√	√
SNP	√	√	√	√	−
Gene	√	√	√	√	−
MR exposure	√	−	√	−	√
MR outcome	√	−	√	−	√
**Output**					
Data table	√	√	√	√	√
Interactive network	+++	+++	++	++	−
Forest plot	√	−	−	−	√
Scatter plot	√	−	−	−	√
Funnel plot	√	−	−	−	√
**Functions and resources**					
Mendelian randomization	√	−	* √	−	√
Exposure (metabolite)	** 4238 metabolic traits, 65 studies	−	123 metabolic traits, 1 study	−	123 metabolic traits, 1 study
Enrichment analysis	√	√	−	−	−
Pre-computed phenome-wide MR	√	−	√	−	−
Semantic triples evidence	√	−	√	−	−

URL links: EpiGraphDB: https://www.epigraphdb.org/ (accessed on 23 January 2023). The Molecular Human: http://comics.metabolomix.com/ (accessed on 23 January 2023). MR-Base: http://www.mrbase.org/ (accessed 23 January 2023). * EpiGraphDB contains pre-computed MR causal estimates. ** Metabolic trait number includes both metabolites, metabolic features and metabolite ratios based on mGWAS-Explorer 1.0 when the effect size and standard error are available in the summary statistics.

## Data Availability

The data are available from https://www.mgwas.ca (accessed on 1 March 2023).

## Supplementary Materials

The following supporting information can be downloaded at: https://www.mdpi.com/article/10.3390/metabo13070826/s1. Figure S1: assessment of causal effects of arachidonic acid levels on Crohn’s disease: a leave-one-out plot and a funnel plot; Table S1: Mendelian randomization results.
