# Supplementary material for: mGWAS-Explorer 2.0: Causal Analysis and Interpretation of Metabolite–Phenotype Associations
Source: Metabolites. 2023 Jul 5;13(7):826. doi: 10.3390/metabo13070826 (PMC10384390; doi:10.3390/metabo13070826)
Supplement: Supplementary file 1 [file metabolites-13-00826-s001.zip › supplementary_figures.pdf]

(a)

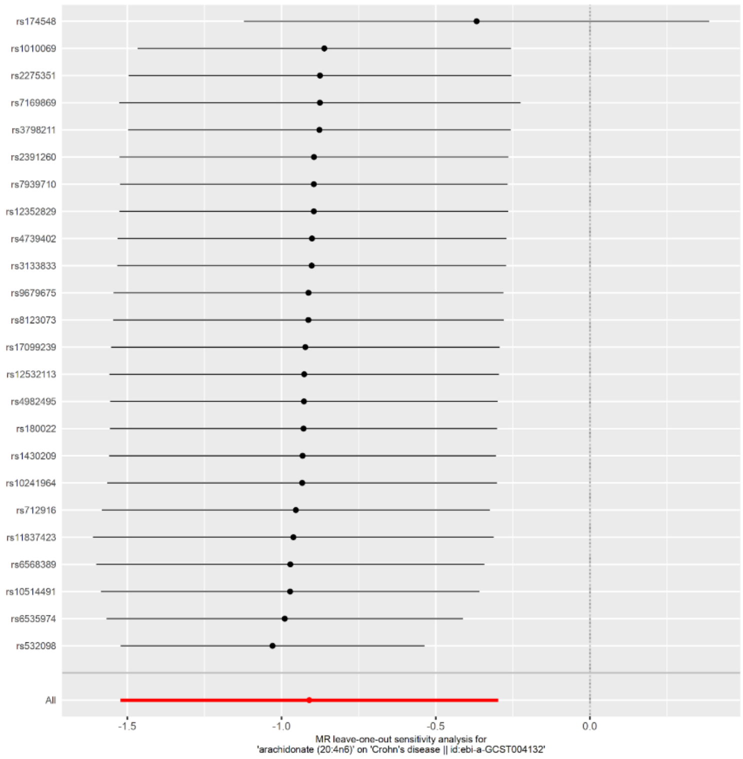
rs174548
rs1010069
rs2275351
rs7169869
rs3798211
rs2391260
rs7939710
rs12352829
rs4739402
rs3133833
rs9679675
rs8123073
rs17099239
rs12532113
rs4982495
rs180022
rs1430209
rs10241964
rs712916
rs11837423
rs6568389
rs10514491
rs6535974
rs532098
All
-1.5
-1.0
-0.5
0.0
MR leave-one-out sensitivity analysis for
'arachidonate (20:4n6)' on 'Crohn's disease || id:ebi-a-GCST004132'

(b)

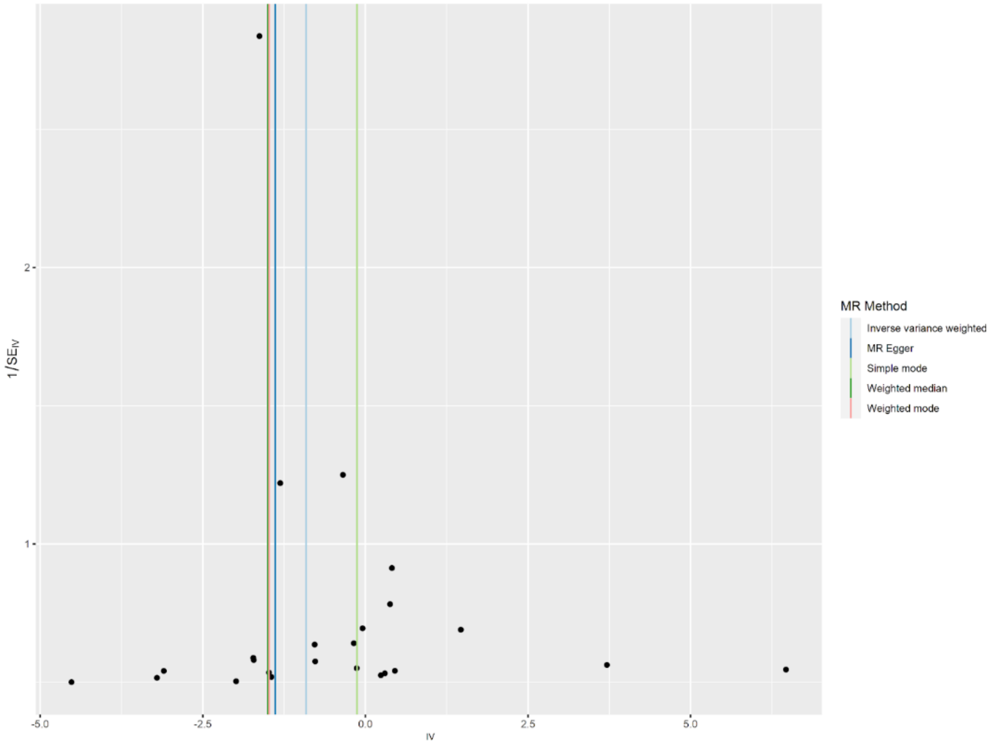
1/SE_IV
2
1
-5.0
-2.5
0.0
2.5
5.0
IV
MR Method
Inverse variance weighted
MR Egger
Simple mode
Weighted median
Weighted mode

Supplementary Figure S1. Assessment of causal effects of arachidonic acid levels on Crohn's disease. (a) a leave-one-out plot, which determine whether a single SNP is having a disproportionately larger impact on an association; (b) a funnel plot, showing the relationships between the causal effect of arachidonic acids on Crohn's disease calculated using each individual SNP as a separate instrument against the inverse of the standard error of the causal estimate.
